# The moderating role of information technology governance in the relationship between board characteristics and continuity management during the Covid-19 pandemic in an emerging economy

**DOI:** 10.1057/s41599-023-01552-x

**Published:** 2023-03-10

**Authors:** Faozi A. Almaqtari, Najib H. S. Farhan, Hamood Mohammed Al-Hattami, Tamer Elsheikh

**Affiliations:** 1 Faculty of Business, Economics and Social Development, Universiti Malaysia Terengganu, Kuala Nerus Terengganu, 21030 Malaysia; 2 Faculty of business studies, Arab Open University, Riyadh, Saudi Arabia; 3 Department of Accounting, Faculty of Commerce and Economic, Hodeidah University, Al Hudaydah, Yemen; 4 Faculty of Commerce, Kafrelsheikh University, Kafr El Sheikh, Egypt

**Keywords:** Library science, Business and management, Finance

## Abstract

The main aim of the current study is to investigate the relationship between governance characteristics, information technology governance, and continuity management during Covid-19 in an emerging economy. The study also examines the moderating role of information technology governance in the relationship between governance characteristics and business continuity management. The quantitative approach is used by utilising a survey questionnaire. A sample of 232 questionnaire surveys has been collected from the board of directors, top and middle management executives, external auditors, information technology experts, and some other respondents. The results were estimated using structural equation modelling. The results indicate that information technology governance has a statistically significant effect on business continuity. Board size, board independence, audit committee independence, audit committee diligence, and external audit have a statistically significant positive effect on information technology governance. Further, the results indicate that information technology governance significantly moderates the effect of board size, board independence, board diligence, audit committee independence, audit committee diligence, and external audit on business continuity. However, information technology governance does not moderate the relationship between board committees and business continuity, which indicates less board involvement in information technology governance. The current research provides insight into the role of information technology governance in business continuity management during crises. The present study provides a unique contribution as it investigates the relationship between corporate governance characteristics, information technology governance, and business continuity management during Covid-19, providing empirical evidence from an emerging country.

## Introduction

The coronavirus pandemic has caused a substitutional business disruption. Some firms may be forced to close permanently due to this disruption (Kaushik & Guleria, 2020). The pandemic created new challenges for global consumers, leading to the use of digital technology (Al Halbusi et al., 2022; Cifuentes-Faura, 2020). It also impacted psychological health and quality of life (Aqeel et al., 2022; Farzadfar et al., 2022; NeJhaddadgar et al., 2022; Yu et al., 2022; Zhou et al., 2022; Su et al., 2022; Hossain et al., 2020; Dong et al., 2021; Nueangnong et al., 2020). The Covid-19 pandemic triggered an economic crisis and a public health emergency, jeopardising energy efficiency consumption, long-term food diversity, and household nutrition security (Zhuang et al., 2022; Zafar et al., 2022; Jiakui et al., 2023; Hossain et al., 2020; Cifuentes-Faura, 2021b). It has consistently influenced environmental behaviour by reducing income and disrupting economies (Geng et al., 2022). Most business operations across industries and sectors, including manufacturing, services, global supply chains, commercials, travelling, hospitality, cargo traffic, tourism, and education, have either halted or slowed dramatically and significantly (Barua, 2020).

Further, as the Covid-19 pandemic spreads, and it is unlikely to unfold, some firms will need years to recover (Kaushik & Guleria, 2020). This increases the business risk and affects business continuity (BC), which is becoming increasingly fragile (Zsidisin et al., 2005). Business continuity management (BCM) seeks to identify these risks to plan for, avoid, or limit them and keep business operations and services running smoothly (Gibb & Buchanan, 2006). Herbane et al. (2004) indicate that BCM is a socio-technical strategy focusing on anticipating potential continuity issues for retaining the organisation’s value. Information technology (IT) is one of the most crucial aspects of BCM. Business enterprises increasingly rely on technology and their ability to integrate IT resources effectively with other organisational and administrative tasks (Lindström et al., 2010; Li et al., 2022).

In Jordan, a survey has been conducted by Kebede et al. (2021), comprising 2039 enterprises from different geographical regions and industries. The survey found that most businesses reported decreasing demand for their products and services, resulting in revenue declines and financial challenges. Mandatory closures harmed half of the surveyed businesses, and closed marketplaces impacted more than a third. Despite government efforts to reduce layoffs and unemployment, one-third of the businesses attributed their layoffs to Covid-19. The tourism industry had the highest percentage of layoffs (48%), followed by construction (45%) and manufacturing (34%). Rent (61%), wages, social security payments (51%), and invoice payments (30%) were the most significant financial burdens on enterprises throughout the pandemic. With regard to the most significant economic impact, eight out of ten businesses reported lower sales, followed by a lack of capital and liquidity issues (six out of ten). Almost half of the respondents said they had lost money. One-quarter of the businesses surveyed had bank loans, and more than one-third had either supplier credit or informal credit from family or friends. One out of every five businesses confirmed that their debt increased. Large and medium-sized businesses, notably those in the manufacturing and construction industries, had the highest debt-to-bank ratio.

Several studies have been conducted to assess various recent issues (Abbas et al., 2019; Azadi et al., 2021; Yao et al., 2022; Yu et al., 2022; Zhou et al., 2022; Li et al., 2022; Zafar et al., 2022). However, these studies did not investigate the effect of IT governance on business continuity. According to Wan and Chan (2008), the BCM approach should include both business and technological elements. The technological component of the BCM improves the firm’s capability to sustain the minimum work requirements in case of a business interruption. Several studies (e.g., Pathak et al., 2020; Al-Hattami et al., 2022; Al-Hattami & Kabra, 2022; Dwivedi et al., 2020; Su et al., 2022) have stressed the importance of IT strategies in achieving corporate success, particularly during pandemics. As a result, to deal with the pandemic, most enterprises were forced to adjust their policies through digitalisation and remote working (Carroll & Conboy, 2020). Some businesses have begun to operate via the ‘Work from Home’ mode, utilising disruptive technology to deal with the economic disruption that happened because of Covid-19 (Kaushik & Guleria, 2020; Dwivedi et al., 2020).

Zhang et al. (2016) indicate that, based on strategic choice theory, IT governance is significantly influenced by corporate governance characteristics (CGC), including board involvement, which is reflected by board knowledge of IT (Jewer, McKay, 2012). In the same context, Zhang et al. (2016) indicate that board involvement in IT is more likely to be evaluated by external auditors and audit committees. The ability of businesses to combine IT and other corporate resources must be improved, especially when the board is made up primarily of independent directors who are probably to develop a more resilient IT competence. Therefore, the current research proposes that corporate governance characteristics alone are insufficient to combat the business disruptions caused by Covid-19. IT governance is needed to maintain BC and avoid business disruptions caused by Covid-19.

Based on this background, two central questions form the main focus of the present study: (1) To what extent did CGC influence BC during the Covid-19 pandemic? (2) Did IT governance moderate the effect of CGC on BC during the Covid-19 pandemic? Accordingly, the current study is motivated by the Covid-19 pandemic’s consequences, due to which all businesses and enterprises were negatively impacted and business operations were interrupted. Therefore, we assume that corporate governance attributes alone are not efficient enough to run business operations smoothly during the crisis. Hence, IT governance can play an effective role in enhancing business efficiency and avoiding business interruptions during crises, contributing to an efficient, holistic, and strategic BC process.

Therefore, this study contributes to the strand literature on CGC, IT governance, and BCM in several ways. First, it provides empirical evidence from an emerging country on the relationship between CGC, IT governance, and BCM. Second, it assesses the mediating role of IT governance in the relationship between CGC and BCM. We propose that during the Covid-19 crisis, corporate governance mechanisms alone are efficient in managing business interruptions and continuities. Third, there is a serious gap in the strand literature on these issues. Very few studies and limited research have been conducted on IT governance and BCM (e.g., Wan & Chan, 2008, crisis management (e.g., Sahebjamnia et al., 2015), and IT governance (e.g., Järveläinen, 2013; Zhang et al., 2016). However, there is a scarcity of studies investigating this issue in the context of the Covid-19 pandemic. Hence, the current research makes a novel contribution to the state-of-the-art and bridges the gap in prior studies. To the researchers’ knowledge, this is the first study that investigates the role of IT governors in the relationship between corporate governance attributes and BCM. Finally, as a methodological contribution, the present research assesses the perceptions of the board of directors, executives, and other respondents from different sectors during the crisis, providing valuable insights into how businesses managed their business disruptions during Covid-19. The respondents’ responses have been estimated using structural equation modelling, which has high statistical power for providing clear and meaningful findings that can establish a holistic approach and framework to help businesses avoid disruptions. Accordingly, the present study is beneficial and significant for business organisations’ board members, policymakers, IT specialists, and academicians. It offers valuable insights into the influence of IT governance during the crisis and how corporate governance mechanisms can be complemented by IT governance to avoid business disruptions and maintain BC.

The next section discusses the background and hypotheses development; section “Methods” outlines the research method; section “Results” is devoted to the empirical results; section “Discussion and implications” provides discussions, implications, and research limitations.

## Background and hypotheses development

### Covid-19 background

Covid-19, with its various variants, continues to worry the world. The story is that, by the end of 2019, an unwanted guest turned the world upside down. It began precisely in December 2019 when the Chinese government notified the World Health Organization (WHO) about the spread of an unknown disease in Wuhan (Nueangnong et al., 2020; Cifuentes-Faura, 2020; Cifuentes-Faura, 2021a). The disease spread unexpectedly fast worldwide and became a pandemic (Nueangnong et al., 2020). Covid-19 has resulted in a significant short-term economic downturn, the closure of many businesses, the unemployment of tens of millions of people, and other repercussions on commercial activities. Covid-19 is a pandemic wreaking havoc on the global economy and causing massive disruptions to lives and livelihoods. According to many assessments, it is the worst worldwide disaster since World War 2 (Engidaw, 2022; Nueangnong et al., 2020). The disease created significant and massive business and service downtime (Kaushik & Guleria, 2020; Buheji, 2020). To mitigate the spread of the disease, most countries used various regulations, including travel bans, security measures, and social distancing (Fabeil et al., 2020; Nueangnong et al., 2020).

As observed by Barua (2020), Covid-19 presented a dramatic impact on international business and threatened the widespread economic well-being of entire countries to the point where delocalisation is imminent. This includes multiple industries from various sectors, such as distribution networks, transportation and cargo flow, production, commercial operations, academic learning, and tourism. The viral outbreak has brought about catastrophic destruction and company closures. Getting past these challenges will not ensure a prosperous or even a long-term positive future outlook (Donthu & Gustafsson, 2020). This forced scientists and researchers to find a way out of this crisis (Alshebami & Rengarajan, 2020). In light of this, the use of technology, like the Internet, and food and environmental security has been found beneficial to curbing the pandemic (see Al-Hattami, 2021; Cifuentes-Faura, 2020; Su et al., 2022; Jiakui et al., 2023; Zafar et al., 2022; Zhuang et al., 2022; Li et al., 2022; Liu et al., 2022).

### Research background and hypotheses development

Several prior studies have examined BC from various aspects (e.g., Cerullo & Cerullo, 2004; Zsidisin et al., 2005). Further, some studies have been conducted on crisis management (e.g., Torabi et al., 2016; Hazaa et al., 2021; Sahebjamnia et al., 2015; Tosh et al., 2014; Liu et al., 2022). The context of these studies is narrow and limited to some crises other than Covid-19, which has caused massive effects. Furthermore, various studies have examined CGC (e.g., Hashed & Almaqtari, 2020; Youssef & Diab, 2021; Almaqtari & Hashed, et al., 2020; Farhan et al., 2020; Almaqtari & Shamim et al., 2020; Almaqtari & Al-Hattami et al., 2020; Al Maqtari & Farhan et al., 2020). However, no study has linked IT governance, BC, and CGC. While some studies focused on IT in the context of BC (e.g., Gómez et al., 2017; Haouam, 2020; Wahab & Arief, 2015; Järveläinen, 2013), they focused more on information technology than IT governance. Similarly, very few studies have investigated IT governance (e.g., Hamdan et al., 2018); however, they paid more attention to financial issues. In addition, despite some studies on governance characteristics, BC, and IT governance, these studies did not investigate the relationship between them in the context of Covid-19. Accordingly, there is a dearth of studies in the strand literature on the relationship between IT governance and BC during Covid-19.

#### Board characteristics and business continuity management

Gibb and Buchanan (2006) indicated a relationship between BCM and information management; both focused on uncertainty. Bunjongmanomai et al. (2020) investigated the relationship between corporate governance and BC during Covid-19. They report that BCM is considered a vital element of corporate governance that functions to control disruptive incidents. Similarly, Tosh et al. (2014) provided evidence of the relationship between hospitals’ ITG and BCP during Covid-19. They revealed that IT readiness is essential for connection and operations. They also contended that information technology improves hospital preparation, business operations, and the health system as a whole. As a result, a thorough BCP describing IT systems and infrastructures should be prepared. IT preparedness is critical for hospitals and health systems to maintain their operations networks, operate health and administrative information systems, and have sufficient capacity to restore and support health and administrative operations (Tosh et al., 2014).

Numerous recent studies have investigated BCM in various contexts (Aragão & Fontana, 2022; Ewertowski, 2022; Ino and Watanabe, 2022; Kaur et al., 2022; Kosieradzka et al., 2022; Le and Nguyen, 2022; Robertson et al., 2022; Singh and Jain, 2022). The researchers agree that BC is critical for business organisations during disruptive incidents. Lindström et al. (2010) indicated that IT and information security are essential elements of BCP. Tammineedi (2010) stated that a dedicated BCM team is necessary in the case of business disruption to enable the efficient continuation of business activities. Experts in business risks, IT, and organisational activities should be included in the team. Moreover, critical business functions should collaborate with their IT application support teams to develop a comprehensive and consistent BCP. The BCM group has to be organised in a hierarchical framework. The group should consist of individuals with relevant expertise and credentials to address pandemic-related constraints.

Several experiences have been provided by different studies on BCM during crises. For example, Goromaru et al. (2021) reported that Covid-19 has severely influenced many enterprises. Hence, any enterprise should establish BCP. The pandemic left consequences that will continue over the coming years. Consequently, experiences from this pandemic should be learned to avoid the negative effects and apply these lessons to future BCP. BCP is recommended during a pandemic to increase elasticity in the face of uncertain future hazards. In another context, Meechang et al. (2021) indicated that flood disasters in Thailand prompted the adoption of BC management, prompting enterprises to consider their long-term viability and sustainability.

The threat of business disruption grows as firms become more reliant on IT infrastructure. The BCP strategy seeks to mitigate the impact of any major business system failures (Cerullo and Cerullo, 2004). Ostadi et al. (2021) reveal that BCM is a complete strategy for identifying risks and mitigating their effects on an organisation’s operations. Product recovery and resource allocation following disruptive incidents are essential components of BCM. Organisations should prioritise resource allocation for restarting activities, minimising expenses, and returning operations to a tolerable level, so that disruptive incidents do not impede important activities. Therefore, the following hypothesis has been framed:

*H*_*0*_*1. There is no significant impact of CGC on BCM during the Covid-19 pandemic*.

#### Corporate governance characteristics and IT governance

ITG exists at the three hierarchical levels of an organisation involving the board and senior executives. The board of directors and the top management develop an IT strategy that will be implemented at the level of operations, including IT management in a practical sense (Haes & Grembergen, 2009). Institute (2003) indicates that developing an IT project charter is the duty of the board of corporate directors and top management. Gómez et al. (2017) argue that one of the board’s responsibilities is to anticipate and monitor IT deployment strategies to increase business value by providing faster resolutions and higher-quality product delivery. They also show that ITG is flawed and externalised if there is no effective board involvement and if the board believes that ITG is not a major aspect of corporate governance.

Haes and Grembergen (2005) highlight that ITG exists at several heretical levels within an organisation. It is situated at the strategic, management, and operational levels. These levels respectively represent the board of directors, C-suite, senior management, operational IT, and business management, where they involve, develop, and implement ITG strategy. According to Moeller (2013), developing high-level courses of action and conducting a comprehensive examination of overall corporate behaviour in light of ITG are the board’s and audit committee’s primary roles for setting the tone at the top. Risk mitigation, disclosure, and accountability all fall under information security (IS). Posthumus and Solms (2004) argue that the senior executive and the board of directors have a corporate management responsibility to deal with (IS). Hamdan et al. (2018) suggested a paradigm for interlocking boards and ITG in Jordan. According to the findings, ITG is a critical practice in the development and structuring of the board, i.e., it is important to connect the board of directors with competent managers with practical expertise in information systems. In another context, according to Lunardi et al. (2014) paper, there are indicators that ITG policies can help firms manage and utilise technology compared to those who do not employ them. Consequently, the subsequent hypothesis has been formulated:

*H*_*0*_*2. There is no significant impact of CGC on ITG during the Covid-19 pandemic*.

#### Corporate governance characteristics, IT governance, and business continuity

Covid-19 has put forward unique challenges in different aspects of life (Aqeel et al., 2021; Maqsood et al., 2021; Rahmat et al., 2018; Zhou et al., 2022). Since the break out of the deadly virus, Covid-19 spreads fear among people at the social level. Therefore, it is critical to implement appropriate mental and physical health prevention measures, particularly in less developed countries. Accordingly, social media could play a significant role in this regard (Abbas et al., 2019; Yu et al., 2022). People who were quarantined due to the spread of the disease could meet online (Yu et al., 2022). This is not limited to communication needs but also educational needs (Azadi et al., 2021; Maqsood et al., 2021; Rahmat et al., 2018; Yao et al., 2022). Moreover, business activities (Aqeel et al., 2021; Yu et al., 2022; Zhou et al., 2022) and the overall smoothness of life have raised the importance of technology to satisfy these needs.

A number of prior studies have assessed CGC throughout the viral pestilence (Covid19) (Elmarzouky et al., 2021; Jebran, Chen (2020); Koutoupis et al., 2021; Li et al., 2021; Xuguang et al., 2021; Zattoni and Pugliese, 2021). However, these studies did not investigate the relationship between CGC and ITG, especially during the pandemic. Several studies also focused on the importance of IT management in governance frameworks. For example, Korac‐Kakabadse and Kakabadse (2001) indicated that ITG is a significant component of governance characteristics that aims to establish associations and alignment among business processes. ITG is thus a significant element of an organisations’ corporate governance model because it introduces critical strategic plan measures that focus on IT strategy alignment. As ITG is initiated by corporate governance, the relationship between the two becomes clear (Dittmeier, 2011).

One of the most commonly used frameworks of ITG is the “Control Objectives for Information and Related Technology” (COBIT) framework (Simonsson et al., 2010; Lunardi et al., 2014). The COBIT framework considers the executive board, the chief executive director, and a few other elements as essential intra-stakeholders. Further, it emphasises the necessity of ITG and the effect of a dynamic and autonomous board of directors as a crucial aspect of the Committee of Sponsoring Organizations (COSCO) control environment (Moeller, 2013).

The board of directors’ size, insiders’ ratio, and board members’ experience in IT significantly influence the extent of the board’s involvement in IT governance (Jewer & McKay, 2012). Nevertheless, Huff et al. (2006), Bart and Turel (2010), and Andriole (2009) indicate that boardrooms have less expertise in IT governance in most cases. According to Peterson (2004), IT governance has to comprise an IT organisation structure, a ‘Chief Information Officer, an IT strategy committee, and an IT steering committee.’ Haes and Grembergen (2009) note that the IT governance structure should include an IT strategy committee at the board level to guarantee that IT is a regular agenda item for the board of directors. Furthermore, to assess the value and risk of IT, the board of directors would need to include IT expertise and experience, as well as an independent IT audit committee. The promotion, direction, and management of IT governance procedures are within the purview of the IT governance officer. At the executive or senior management level, the IT ‘steering committee’ should be accessible to determine the business priorities for IT investments. Importantly, Haes and Grembergen (2005) examined IT governance through interviews and reports. They claim that consultants, rather than board members, steer IT governance issues. As a result, the following hypothesis has been proposed:

*H*_*0*_*3. There is no significant moderation effect of ITG on the relationship between CGC and BCM during the Covid-19 pandemic*.

## Methods

### Research framework

**Fig. 1:**
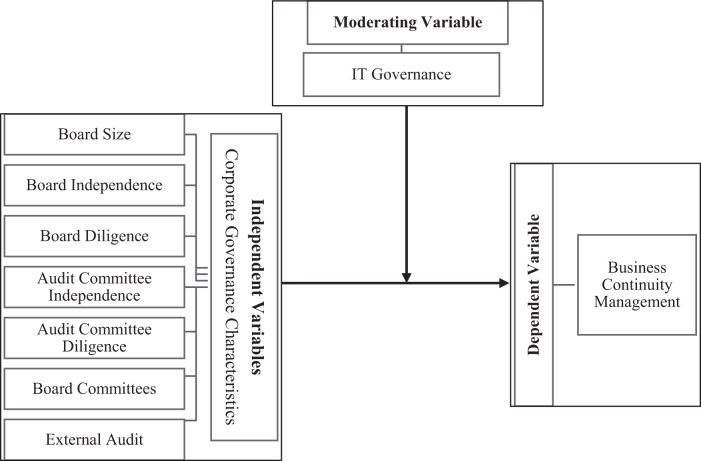
Research framework. The research framework comprises three main variables: CGC, BCM, and ITG. CGCs are considered independent variables measured by board size, board independence, board diligence, audit committee independence, audit committee diligence, board committees, and external audit. BCM is treated as the independent variable, and ITG is a moderating variable.

Figure 1 illustrates the research framework.

### Data and sample

The current study’s target research population includes all Jordanian businesses from different sectors operating in Jordan. We targeted different categories, including board members, senior executives, auditors, and IT assistants of various generations. The data for the study was collected through a snowball sampling procedure. Different researchers confirm that the snowballing sampling method is effective and appropriate for multivariate data processing and estimating the results (Agyekum et al., 2021; Chan, 2020; Faugere and Stul, 2021; Noy, 2008; Wang et al., 2019; Wright and Stein, 2004). At the initial stage, we explored the required minimum sample size to estimate the results. Many studies provide formulas and rules of thumb to calculate the sample size required to estimate the results (Bollen, 1989; Christopher Westland, 2010; Long et al., 1990). Following these studies and based on PLS path modelling and the number of latent and observed variables, we calculated the minimum sample size using free online statistical software. The sample is calculated based on an anticipated effect size of 0.3, a desired statistical power level of 0.8, nine latent variables, and 37 observed variables with a probability level of 1%. This gives a minimum sample size of 184 respondents. In addition, we used G-Power software to determine the required minimum sample, which yielded a minimum sample size of 160 respondents.

However, the study collected 232 surveys through an online questionnaire survey via Google Docs using convenience sampling. The online survey was administered through several social media platforms (e.g., Facebook, WhatsApp, and e-mails) to increase the possibility of data collection. All questions were made mandatory to avoid incomplete forms or missing data. In the same context, the survey is based on closed-ended questions (Westland, 2014), where all items were made with respondent-friendly statements to increase the response rate and avoid poor-quality responses (Tarran, 2010). Moreover, the response rate is enhanced by sending a short letter to targeted respondents through distribution platforms. Moreover, brevity is also used, which yielded an increase of 20% in the response rate.

Therefore, 232 surveys were collected and considered the final sample for the present study. Table 1 provides the sampling and sample adequacy. The results show that the final sample is 232. As the Kaiser-Meyer-Olkin Measure of Sampling Adequacy” value is greater than 0.7, this sample is considered statistically adequate for estimating the results. Further, this test shows high significance at the level of 1% (*P*-value = 0.000, <0.01), indicating the suitability and adequacy of the sample. Further, the fitness of the factor analysis is indicated by Bartlett’s test, which has a value of 7956.149. Consistently, the degree of freedom is 741, indicating an appropriate estimation of factor analysis.

**Table 1:** Sampling adequacy test.

Particulars	No.
Total number of completed surveys (Online)	232
The number of incomplete surveys	(0)
Total number of questionnaire forms processed	232
**KMO and Bartlett’s test**	
“Kaiser-Meyer-Olkin Measure of Sampling Adequacy”.	0.963
“Bartlett’s Test of Sphericity”	
Approx. Chi-Square	7956.149
Degree of freedom	741
Sig.	0.000

### Research instrument

The present study utilises an online questionnaire survey distributed to board members, senior executives, auditors, and IT assistants from various sectors of Jordanian organisations. The questionnaire survey consists of thirty-nine items based on a thorough literature review. A 5-point Likert scale ranging from 1 (strongly disagree) to 5 (strongly agree) was utilised to measure and assess the respondents’ perceptions. The questionnaire was divided into nine dimensions. Table 2 below provides the measurement scales along with the operational definitions of the variables.

**Table 2:** Operational definition of the variables.

Nature	Variable	Acronym	Measurement	Evidence
Independent variables	Board Size	BSIZE	4 statements	(Almaqtari et al., 2022) (Al-Thuneibat et al., 2016) (Nalukenge et al., 2017)
	Board Independence	BIND	4 statements	
	Board Meetings	BMET	4 statements	
	Audit Committee Independence	ACIND	4 statements	
	Audit Committee Diligence	ACMET	3 statements	
	Board Committees	BCOM	3 statements	
	External Audit	AUDIT	5 statements	
Moderating variable	ITG	ITGOV	4 statements	(Al-Zwyalif, 2013) (Vugec et al., 2017) (Mushtaque et al., 2014)
Dependent variable	Business Continuity	BC	4 statements	(Järveläinen, 2013) (Järveläinen, 2012) (Rebmann et al., 2013) (Tammineedi, 2010)

## Results

### Sample demographic analysis

Table 3 shows the demographic characteristics of the participants. The findings show that gender distribution has 59 percent for males and 41 percent for females. A sizable proportion of respondents (72%) were under the age of 40 (37% and 35% from the under-40 age groups, respectively). In addition, 52% of the participants held an undergraduate degree, whereas only 45% held a higher education degree (24% were PG holders and 21% were Ph.D. holders). The results also show that while 41 percent of the respondents had less than five years of experience, 27 percent had six to ten years. Similarly, the results indicate that 22% of the respondents had eleven to fifteen years of working experience, compared to 10% with more than fifteen years of working experience.

**Table 3:** Respondents’ profile.

Demographics	Categories	Frequency	Percentage
Gender	Male	136	59%
	Female	96	41%
Age	Less than 30	85	37%
	30–40	80	35%
	41:51	49	21%
	Above 50	18	8%
Qualification	Less than UG	7	3%
	UG	121	52%
	PG	56	24%
	Ph.D	48	21%
Experience	Up to 5 Years	95	41%
	6:10	62	27%
	11:15	51	22%
	Above 15	24	10%

### Measurement model

Several studies have addressed the choice of PLS estimation based on its pros (Al-Hattami et al., 2021; Balta et al., 2020; Banerjee, 2022; Chin, 2010; Rostamzadeh et al., 2021; Shanmugapriya and Subramanian, 2016; Westland, 2014; Al-Hattami, 2022; Al-Hattami, 2023; Zafar et al., 2022). PLS modelling is commonly used among researchers due to several advantages (Hair et al., 2013; Henseler and Sarstedt, 2013). For example, PLS path modelling can be used to estimate associations between latent variables with a variety of indicators, even with a small sample size. The PLS path modelling approach uses ordinary least squares regressions to estimate sample sizes for various components of the focused path model. As a result, sample size requirements are scarcely affected by the complexity of the overall model.

SEM-PLS is an appropriate technique for assessing complicated models that attempt to anticipate associations between research variables (Memon et al., 2017). PLS-SEM can be used to forecast and evaluate key target constructions as well as identify key driver constructs. The reasons for using PLS-SEM include data characteristics such as small sample size and non-normal data. Hair et al. (2019) suggest that there are multiple reasons for PLS estimation: (a) small sample size; (b) models with formatively specified constructs; (c) PLS-SEM is preferable over regression analysis when estimating mediation; (d) researchers should use the two-stage approach to moderator analysis; (e) it is not necessary to estimate a PLS model’s goodness-of-fit.

Accordingly, the present study uses Smart PLS3 software to conduct confirmatory factor analysis, validity, reliability, and structural equation modelling for hypotheses testing. This approach is motivated by similar prior studies (Alsmairat et al., 2018; Awawdeh et al. (2021); Elgharbawy and Abdel-Kader, 2016; Thaker et al., 2022; Wang et al., 2022; Wijethilake, 2017; Zafar et al., 2022). For a more rigorous estimation of the results, SPSS software version 23 was used to conduct exploratory factor analysis and reliability analysis of the measurement model. This is also motivated by Balta et al. (2020), who conducted a study using both SPSS and PLS. The current study also used SPSS to filter the data and assess several assumptions and issues, including residuals, outliers, normality, and multicollinearity.

### Exploratory factor analysis (EFA)

The results in Table 4 provide EFA results. EFA using SPSS 23 was conducted to determine whether the data was sufficient to assess a latent variable network model. The results provide the factor loading values for each indicator, which are greater than 0.40. Further, the results present the total variance, which shows the eigenvalues for the yielded latent variables. Moreover, the findings provide reliability values based on Cronbach’s alpha values. Some items were deleted throughout the exploratory factor analysis due to low factor loadings (0.40) or cross-loadings. Reliability analysis (i.e., Cronbach’s alpha) of the extracted factors was also conducted to ensure that each observed variable has a value greater than 0.70 (Akter et al., 2013).

**Table 4:** Exploratory factor analysis.

Items	Factor loadings	CA	Total variance explained (initial eigenvalues)			Total rotation sums of squared loadings
			Total	% of Variance	Cumulative %	
BSIZE1	0.769	0.891	6.455	17.445	17.445	3.589
BSIZE2	0.867	0.891				
BSIZE3	0.838	0.893				
BSIZE4	0.824	0.891				
BSIZE5	0.733	0.892				
BIND1	0.673	0.894	5.663	15.305	32.75	3.4
BIND2	0.728	0.893				
BIND3	0.68	0.892				
BIND4	0.548	0.892				
BDEL1	0.787	0.891	2.897	7.83	40.58	3.192
BDEL2	0.796	0.892				
BDEL3	0.699	0.891				
BDEL4	0.712	0.892				
BDEL5	0.627	0.891				
ACIND1	0.731	0.892	2.077	5.614	46.194	2.493
ACIND2	0.745	0.892				
ACIND3	0.716	0.892				
ACIND4	0.693	0.892				
ACDEL1	0.792	0.891	1.765	4.771	50.964	2.45
ACDEL2	0.793	0.891				
ACDEL3	0.789	0.89				
BCOM1	0.711	0.892	1.631	4.408	55.372	2.383
BCOM2	0.798	0.892				
BCOM3	0.794	0.891				
AUDIT1	0.806	0.893	1.274	3.444	58.816	2.283
AUDIT2	0.785	0.893				
AUDIT3	0.727	0.892				
AUDIT4	0.699	0.892				
AUDIT5	0.473	0.892				
ITGOV1	0.63	0.894	1.223	3.304	62.12	2.253
ITGOV2	0.733	0.893				
ITGOV3	0.65	0.891				
ITGOV4	0.679	0.89				
BC1	0.566	0.892	1.168	3.158	65.278	2.109
BC2	0.68	0.891				
BC3	0.799	0.892				
BC4	0.721	0.893				

### Confirmatory factor analysis

Table 5 demonstrates the results of confirmatory factor analysis using PLS. The results provide the mean values and standard deviation for each item used to measure each construct. Further, the results give the measurement model in the form of factor loadings, Cronbach’s alpha, composite reliability (CR), and average variance extracted (AVE). Compared to EFA results, it is clear that two items have been deleted: BSIZE5 and BDEL5. The factor loading for these items was <0.40.

**Table 5:** Reliability and validity.

Variables	Acronym	Factor loading	CA	rho_A	CR	AVE
BSIZE	BSIZE 1	0.784	0.877	0.878	0.877	0.641
	BSIZE 2	0.789				
	BSIZE 3	0.801				
	BSIZE 4	0.827				
BIND	BIND 1	0.86	0.92	0.92	0.92	0.741
	BIND 2	0.846				
	BIND 3	0.883				
	BIND 4	0.856				
BDEL	BDEL 1	0.851	0.844	0.869	0.851	0.595
	BDEL 2	0.845				
	BDEL 3	0.795				
	BDEL 4	0.557				
ACIND	ACIND 1	0.832	0.889	0.89	0.889	0.667
	ACIND 2	0.829				
	ACIND 3	0.779				
	ACIND 4	0.826				
ACDEL	ACDEL 1	0.848	0.823	0.83	0.826	0.613
	ACDEL 2	0.735				
	ACDEL3	0.762				
BCOM	BCOM 1	0.808	0.833	0.836	0.834	0.626
	BCOM 2	0.819				
	BCOM 3	0.744				
AUDIT	AUDIT 1	0.853	0.907	0.908	0.907	0.663
	AUDIT 2	0.776				
	AUDIT 3	0.843				
	AUDIT 4	0.794				
	AUDIT 5	0.801				
ITGOV	ITGOV 1	0.863	0.897	0.9	0.897	0.685
	ITGOV 2	0.883				
	ITGOV 3	0.791				
	ITGOV 4	0.769				
BC	BC 1	0.845	0.888	0.89	0.889	0.667
	BC 2	0.827				
	BC 3	0.772				
	BC 4	0.821				

Note: BSIZE is Board Size, BIND is Board Independence, BMET is Board Meetings, ACIND is Audit Committee Independence, ACMET is Audit Committee Meetings, BCOM is Board Committees, AUDIT is External Audit, ITGOV is ITG, and BC is Business Continuity. CA is Cronbach’s Alpha, CVR is Composite Reliability, CR is Average Variance Extracted.

Based on the findings, it can be deduced that the factor loadings of the items have coefficients between 0.55 and 0.88. These values are higher than the acceptable criterion value (0.60) suggested by Chin (2010). CR values range between 0.82 and 0.92. These values indicate how well each construct’s components reflect the latent construct.

**Fig. 2:**
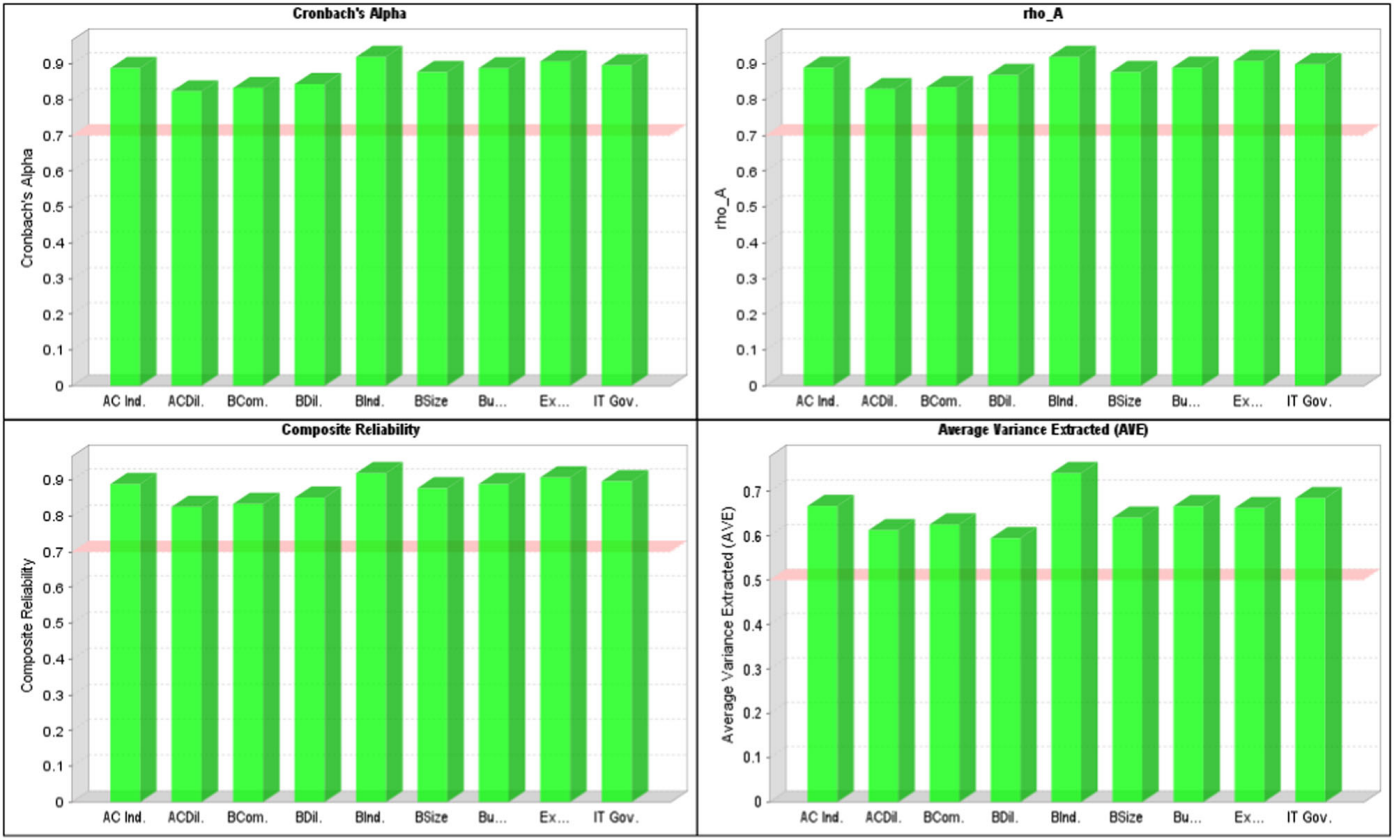
Reliability and validity. This figure shows the values of CA, Roh_A, AVE, and CR. All values are higher than the criterion values, exceeding the lowest value line.

**Fig. 3:**
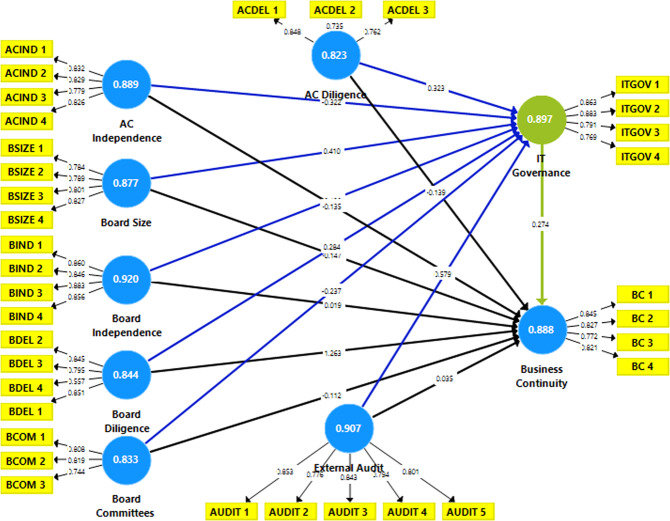
Confirmatory factor analysis. This figure provides the constructs’ confirmatory factor analysis (CFA). The CFA has been estimated based on the conceptual framework presented in Fig. 1 . It delivers the values of the factor loading, validity, and reliability of constructs.

Figure 2 shows the values of CA, Roh_A, AVE, and CR. Figure 3 provides the constructs’ confirmatory factor analysis (CFA).

The findings in Table 6 provide the results of discriminant validity. The results reveal high correlation values corresponding to the same construct, indicating that the items used to measure the construct are suitable and represent the same construct. This is evident as the correlation values of each construct with other constructs provide low correlations, which are less than the self-correlation values of the construct (Fornell and Larcker, 1981).

**Table 6:** Convergent & discriminant validity.

Variables	BSIZE	BIND	BMET	ACIND	ACMET	BCOM	AUDT	ITGOV	BC
BSIZE	**0.855**								
BIND	0.385	**0.898**							
BMET	0.723	0.431	**0.928**						
ACIND	0.753	0.444	0.82	**0.866**					
ACMET	0.67	0.402	0.662	0.679	**0.86**				
BCOM	0.738	0.42	0.697	0.722	0.765	**0.866**			
AUDT	0.781	0.43	0.738	0.779	0.752	0.823	**0.854**		
ITGOV	0.774	0.303	0.674	0.734	0.723	0.747	0.804	**0.874**	
BC	0.755	0.443	0.92	0.799	0.677	0.717	0.777	0.736	**0.866**

Note: BSIZE is Board Size, BIND is Board Independence, BMET is Board Meetings, ACIND is Audit Committee Independence, ACMET is Audit Committee Meetings, BCOM is Board Committees, AUDIT is External Audit, ITGOV is ITG, and BC is Business Continuity.

AVE square root is remarked in bold.

### Structural model

**Fig. 4:**
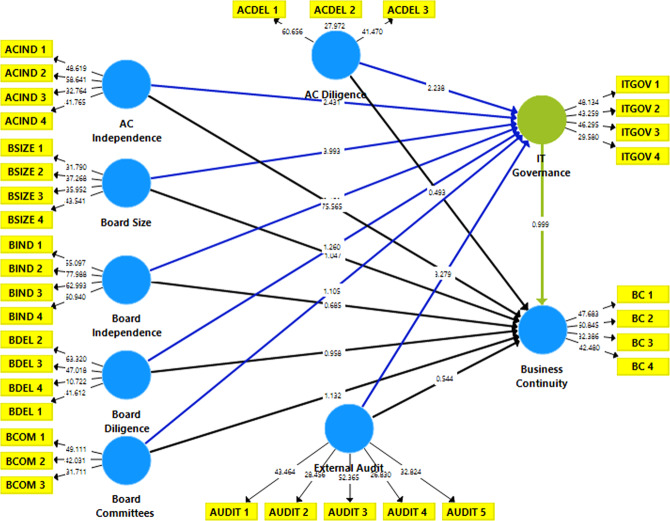
Structural equation model-direct effect. This figure displays the study variables’ hypothesised or predicted structural approach. It provides a direct effect model for the influence of the explanatory variables represented by CGC and ITG on the BC predicted variable.

Figure 4 displays the study variables’ hypothesised or predicted structural approach.

Table 7 provides the estimates for the direct effect. The results in Panel A show that CGCs have an insignificant impact on BC except for ACIND. The results reveal that BSIZE, BIND, BDEL, ACIND, BCOM, and AUDIT exhibited an insignificant effect on BC at any significance level (*P* < 1%, 5%, and 10%) during the Covid-19 pandemic. While board size, board committees, and external audit exhibit statistically significant negative effects, board independence, board diligence, and audit committee diligence show a positive impact. Nonetheless, the evidence reveals that ACIND has a statistically significant positive impact on BC at 1% (*β* = 0.988; *P*-value < 0.01). Notably, the empirical findings show that ITG has a statistically significant effect on BC at 5% (*β* = 0.012; *P*-value < 0.05). The adjusted *R*^2^ is 0.68, meaning the CGC and ITG constitute about 68% of BC. Therefore, H01, which states that “there is no significant effect of CGC on BC,” is rejected in terms of audit committee independence; however, it is accepted concerning BSIZE, BIND, BDEL, ACDEL, AUDIT, and BCOM.

**Table 7:** Structural equation modelling.

Path	Coefficients	Standard errors	*T*-value	Result
Panel A: BC model				
BSIZE → BC	−0.010	0.009	1.091	Accepted
BIND → BC	0.001	0.002	0.703	Accepted
BMET → BC	0.010	0.010	0.988	Accepted
ACIND → BC	0.988	0.012	81.549***	Rejected
ACMET → BC	0.002	0.003	0.515	Accepted
BCOM → BC	−0.018	0.016	1.116	Accepted
AUDT → BC	−0.002	0.004	0.548	Accepted
ITGOV → BC	0.012	0.012	1.055**	Rejected
Panel B: ITGOV model				
BSIZE → ITGOV	0.282	0.074	3.812***	Rejected
BIND → ITGOV	0.110	0.040	2.769***	Rejected
BMET → ITGOV	−0.149	0.121	1.233	Accepted
ACIND → ITGOV	0.267	0.110	2.421**	Rejected
ACMET → ITGOV	0.184	0.077	2.383**	Rejected
BCOM → ITGOV	0.093	0.082	1.133	Rejected
AUDT → ITGOV	0.320	0.101	3.184***	Rejected

Notes: Hypothesis acceptance and rejection criteria are based on 0.01 and 0.10 significance levels, which indicate *** and **.

BSIZE is Board Size, BIND is Board Independence, BMET is Board Meetings, ACIND is Audit Committee Independence, ACMET is Audit Committee Meetings, BCOM is Board Committees, AUDIT is External Audit, ITGOV is ITG, and BC is Business Continuity.

Panel B results for the IT governance model show that board size has a statistically significant positive effect on IT governance at the 1% level (*β* = 3.812; *P*-value < 0.01). The results also show that board independence has a statistically significant positive effect on IT governance at 1% (*β* = 2.769; *P*-value < 0.01). However, the findings indicate that board diligence has an insignificant negative effect on IT governance (*β* = −1.233; *P*-value > 0.10). Further, they reveal that audit committee characteristics represented by audit committee independence and diligence have a statistically significant positive effect on IT governance at the level of 5% (*P*-value < 0.01). In the same context, the results show that board committees have an insignificant positive effect on IT governance (*β* = 1.133; *P*-value > 0.10). Furthermore, the findings indicate that external audit has a statistically significant positive effect on IT governance at 1% (*β* = 3.184; *P*-value < 0.01). The adjusted *R*^2^ has a 0.88 score, indicating that CGC explains about 88% of the variability of IT governance. Hence, H_0_2, which states “there is no significant effect of CGC on IT governance,” is rejected in terms of board size, board independence, audit committee independence, audit committee diligence, and external audit”. However, it is accepted in the context of board diligence and board committees.

### The moderating effect of ITG

**Fig. 5:**
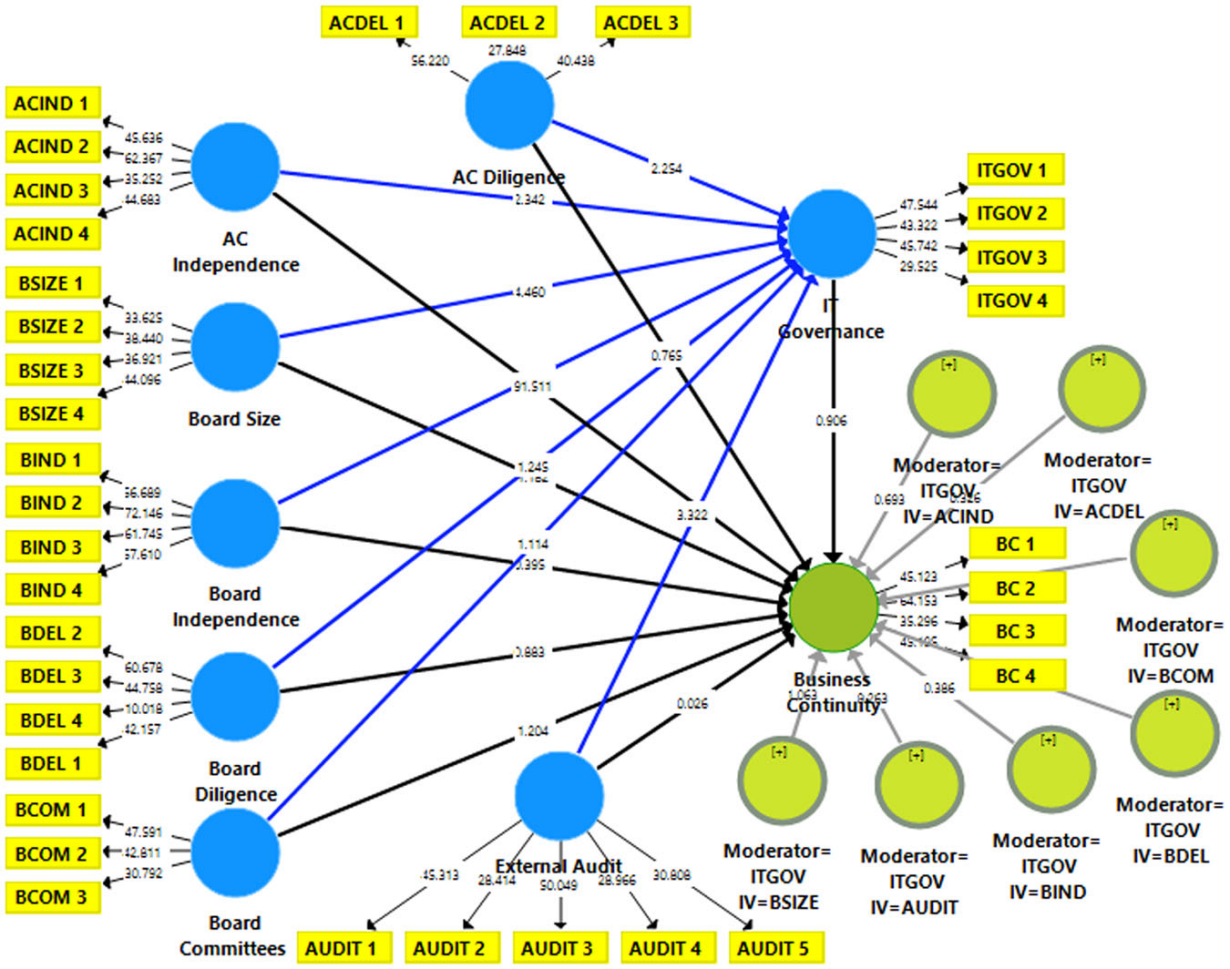
SEM model-moderation effect. This figure presents structural equation modelling for the moderating effect of IT governance on the relationship between governance mechanisms and BC. IT governance has been considered a moderating variable that moderates the relationship between CGC and BC.

Figure 5 presents structural equation modelling for the moderating effect of IT governance on the relationship between governance mechanisms and BC.

Table 8 shows the moderating impact of IT governance results on the association between governance attributes and BC. The results in Panel A are consistent with the findings provided in Table 7, Panel A. The study reveals that CGCs have an insignificant impact on BC except for audit committee independence, which exhibits a statistically significant impact on BC. The study findings also show that IT governance has a statistically significant effect on BC at the level of 5% (*β* = 0.010; *P*-value < 0.05). In addition, Panel B reveals findings similar to those presented in Table 7 and Panel B. The results found that board size, board independence, and external audit have a statistically significant positive effect on IT governance at 1% (*P*-value < 0.01). Further, the findings reveal that audit committee characteristics represented by audit committee independence and diligence have a statistically significant positive effect on IT governance at the level of 5% (*P*-value < 0.01). However, the results indicate that board diligence and board committees exhibit an insignificant negative effect on IT governance (*P*-value > 0.10).

**Table 8:** The moderating role of ITG.

Path	Coefficients	Standard errors	*T*-value	Result
Panel A: Direct Effect- BC Model				
BSIZE → BC	0.017	0.014	1.271	Accepted
BIND → BC	0.001	0.002	0.439	Accepted
BMET → BC	0.007	0.007	0.903	Accepted
ACIND → BC	0.991	0.010	96.213***	Rejected
ACMET → BC	−0.005	0.006	0.865	Accepted
BCOM → BC	−0.020	0.016	1.294	Accepted
AUDT → BC	0.000	0.006	0.024	Accepted
ITGOV → BC	0.010	0.010	1.047**	Rejected
Panel B: direct effect- ITGOV model				
BSIZE → ITGOV	0.282	0.069	4.101***	Rejected
BIND → ITGOV	−0.110	0.038	2.865***	Rejected
BMET → ITGOV	−0.149	0.123	1.214	Accepted
ACIND → ITGOV	0.267	0.117	2.284**	Rejected
ACMET → ITGOV	0.184	0.081	2.268**	Rejected
BCOM → ITGOV	0.093	0.083	1.115	Accepted
AUDT → ITGOV	0.320	0.102	3.155***	Rejected
Panel C: indirect effect -moderation effect				
BSIZE > Moderator ITGOV > BC	−0.019	0.017	1.138***	Rejected
BIND > Moderator ITGOV > BC	0.001	0.003	0.383***	Rejected
BMET > Moderator ITGOV > BC	0.010	0.011	0.912*	Rejected
ACIND > Moderator ITGOV > BC	0.008	0.011	0.737***	Rejected
ACMET > Moderator ITGOV Business > Continuity	0.002	0.004	0.362**	Rejected
BCOM > Moderator ITGOV > BC	−0.019	0.017	1.144	Accepted
AUDT > Moderator ITGOV > BC	0.001	0.005	0.277**	Rejected

Notes: Hypothesis acceptance and rejection criteria are based on 0.01, 0.05, and 0.10 significance, which indicate ***, **, and *, respectively.

BSIZE is Board Size, BIND is Board Independence, BMET is Board Meetings, ACIND is Audit Committee Independence, ACMET is Audit Committee Meetings, BCOM is Board Committees, AUDIT is External Audit, ITGOV is ITG, and BC is Business Continuity.

In terms of the moderating effect of IT governance on the relationship between governance mechanisms and BC, Panel C’s findings indicate that IT governance significantly moderates the effect of board size on BC (*P*-value < 0.01). However, this moderating effect is negative (*β* = −0.019), indicating that board size negatively moderates the IT governance’s effect on BC. This could be due to the large board size, which may negatively affect the impact of IT governance on BC. The outcomes further outline that board independence has a statistically positive (*β* = 0.001; *P*-value < 0.01) moderating impact on the relationship between IT governance and BC. This indicates that board independence has a positive monitoring role that significantly strengthens the bearing of IT governance on BC. The respondents perceived that board independence plays a significant and effective monitoring role in IT governance, contributing to a better BCM.

The research found that board diligence significantly and positively (*β* = 0.010) moderates the effect of IT governance on BC. However, this effect is weak at 10% (*P*-value < 0.10). This could be attributed to the fact that board meetings strengthen the efficiency of IT governance. However, the respondents perceive that board meetings do not strongly moderate BC. This could be because all companies conducted their meetings virtually during Covid-19, which negatively affected the role of board diligence in the relationship between IT governance and BC. The outcomes also reveal that audit committee independence and diligence have a statistically significant positive moderating impact on the relationship between IT governance and BC. At the same time, audit committee independence has a significant impact at a 1% level (*P*-value < 0.01), and diligence has a statistically significant effect at the level of 5% (*P*-value < 0.05). This implies that audit committees have a positive monitoring role that strengthens the relationship between IT governance and BC.

The findings clarify that board committees have a statistically insignificant (*P*-value > 0.10) moderating impact on the relationship between IT governance and BC. The negative coefficient (*β* = −0.019) indicates that this effect is negative but statistically insignificant. Finally, external audit exhibits a statistically significant positive moderating effect of 5% (*β* = 0.001, *P*-value < 0.05) on the relationship between IT governance and BC. This leads to rejecting H03, which states that “there is no significant moderating effect of IT governance on the relationship between CGC and BC.” Therefore, H03 is partially rejected in terms of board size, board independence, board diligence, audit committee independence, audit committee diligence, and external audit; however, it is accepted in the context of board committees.

## Discussion and implications

### Summary of findings

The purpose of this study was to look into the impact of governance characteristics and IT governance on continuity management during Covid-19. The study also examined the moderating role of IT governance in the relationship between governance characteristics and BCM. A quantitative approach was used by utilising a survey questionnaire. A total of 232 questionnaire surveys were received from the board of directors, top and middle management executives, external auditors, IT experts, and some other respondents in Jordan. The study used an online questionnaire survey based on a 5-point Likert scale as the research instrument to collect the data. Finally, factor analysis and structural equation modelling were used to estimate the results.

The outcomes revealed that CGCs have an insignificant impact on BC except for audit committee independence, which exhibits a statistically significant effect on BC. The results also revealed that IT governance has a statistically significant effect on BC. The study found that board size, board independence, and external audit have a statistically significant positive impact on IT governance. Furthermore, the findings revealed that audit committee characteristics, represented by audit committee independence and diligence, have a statistically significant positive effect on IT governance. However, board diligence and board committees exhibited an insignificant negative effect on IT governance.

Regarding the moderating impact of IT governance on the relationship between governance mechanisms and BC, the results reported that IT governance significantly moderates the effect of board size on BC. However, this moderating effect is negative, indicating that board size moderates the effect of IT governance on BC negatively. The outcomes also show that board independence has a statistically significant positive moderating impact on the relationship between IT governance and BC. The results found that board diligence significantly and positively moderates the effect of IT governance on BC. However, this effect is weak at 10% (*P*-value < 0.10). In the same context, the study shows that audit committee independence and diligence have a statistically significant positive moderating impact on the relationship between IT governance and BC. Furthermore, the findings show that IT governance does not moderate the relationship between board committees and the BC. Finally, external audit exhibits a statistically significant positive moderating effect on the relationship between IT governance and BC.

The research at hand provides insight into the role of IT governance in BCM during crises. It offers a unique contribution as it investigates the relationship between CGC, IT governance, and BCM during the Covid-19 era in an emerging country. The study provides empirical evidence from an emerging country on the relationship between CGC, IT governance, and BCM. Moreover, the present study makes a unique and novel contribution to investigating a critical issue encountered by all businesses during Covid-19, that affected business operations. To the researchers’ knowledge, this is the first study on the role of IT governors in the relationship between corporate governance attributes and BCM. Therefore, the present study also contributes to the strand of literature and bridges a serious gap. Very few studies and limited research have been conducted on IT governance and BCM. Accordingly, the present study is beneficial and highly important for board members of corporate organisations, stakeholders, regulators, practitioners, and academicians. The study is based on empirical evidence from a developing country. Accordingly, the results of this study have wider practical applications for some other developing nations. It offers insights into using technology-based business during crises for better business continuity.

### Practical implications

Manufacturing industries are facing numerous challenges as a result of the Covid-19 pandemic and changing market demands (Pansare et al., 2022). The Covid-19 pandemic has significantly impacted most manufacturing systems, affecting the supply chain of medicine and other products (Moosavi et al., 2022). Furthermore, the manufacturing industries are struggling to improve performance and re-establish the supply chain in the post-Covid-19 period. To improve performance, current market demands and the post-Covid-19 situation necessitate integrating IT strategies and technological capabilities (Pansare et al., 2022).

The results of the present study report that all CGCs, except for audit committee independence, have an insignificant effect on BC. They also indicate that IT governance has a statistically significant effect on BC. Further, the results found that board size, board independence, audit committee independence, audit committee diligence, and external audit have a statistically significant positive effect on IT governance. However, the results show that board diligence exhibits an insignificant negative impact on IT governance. Overall, the results show that board involvement in IT governance was inefficient during the crisis in Jordan. Consistently, Moeller (2013) indicates that as a fundamental component of the Committee of Sponsoring Organizations (COSCO) control environment, the COBIT framework emphasises the importance of IT governance and the role of an effective and independent board. Thus, board members need to enhance their involvement in IT governance to improve preparedness for any crisis and improve business operations. In this regard, companies, especially board members, are suggested to incorporate both business and technological elements into their BCM process. Moreover, a detailed BC specifying IT systems and infrastructures should be created.

Several studies also emphasise the board’s responsibilities and involvement in monitoring and developing IT governance strategy (e.g., Gómez et al., 2017; Posthumus & Solms, 2004; Hamdan et al., 2018; Moeller, 2013). In addition, many studies report that information technology, including IT governance, is considered one of the prominent elements of a BC plan (Lindström et al., 2010; Korac-Kakabadse & Kakabadse, 2001; Dittmeier, 2011; Haes & Grembergen, 2009; Peterson, 2004). They indicate that IT governance is a significant element of an organisation’s corporate governance model because it introduces critical measures for strategic plans focusing on IT strategy alignment.

The results of the current study exhibit that CGCs have an insignificant effect on BC; however, they indicate a significant effect on IT governance. This could be because several enterprises began adopting IT governance to achieve better alignment in business operations (Haes & Grembergen, 2009). Further, IT governance has been identified as a critical concern for businesses. Companies’ growing interest in the subject is justified by the changing role and relevance of IT within organisations and the need to ensure that it is properly managed. IT governance employs corporate governance concepts to drive and control IT strategically (Lunardi et al., 2014). Therefore, IT governance is high on the agenda nowadays, and many organisations are incorporating its practices into their day-to-day operations (Haes & Grembergen, 2009, Lunardi et al., 2014). Accordingly, business organisations should enhance their IT governance mechanisms as a practical implication. Moreover, board and audit committee members should have capacity programmes that enhance their expertise in IT governance. IT resources should be integrated with other organisational resources in the IT governance of business organisations to provide a competitive advantage (Zhang et al., 2016). This is necessary as a pandemic reaction (Ferreira et al., 2021).

Regarding the moderating effect of IT governance on the relationship between governance mechanisms and BC, the results reported that IT governance significantly moderates the effect of board size, board independence, board diligence, audit committee independence, audit committee diligence, and external audit on BC. However, IT governance does not moderate the relationship between board committees and BC. The current study results are consistent with Lindström et al. (2010), who indicate that IT is one of the key drivers of BC. Further, Tosh et al. (2014) revealed that IT played a significant role in BC planning during the pandemic. Numerous studies have consistently indicated that a BC plan should be designed and implemented to avoid the unintended consequences of disruptive events (e.g., Sahebjamnia et al., 2015; Botha & Solms, 2004; Cerullo & Cerullo, 2004). Similarly, IT governance is necessary to ensure the continuity and recovery of an organisation’s business operations to a predetermined acceptable level after a disruptive event (Tammineedi, 2010; Lindström et al., 2010; Clifton, 2000; Botha & Solms, 2004; Cerullo & Cerullo, 2004).

Accordingly, business organisations should identify possible risks and establish a framework for building response and resilience as part of their business continuity. Business organisations should frame their BC plans as a process of sustaining their business operations and maintaining their continuity following a disruptive event that can impede their goals (Aleksandrova et al., 2018). Any minor disruption can cause irreversible harm to a company’s reputation and public image (Botha & Solms, 2004). Accordingly, a well-designed and efficient pre-crisis BC plan should be designed and implemented (Sahebjamnia et al., 2015). Hence, the results of the current study suggest that BC plan methodology should be developed and implemented to avoid the undesirable consequences of disruptive events (Botha & Solms, 2004; Cerullo & Cerullo, 2004). To this end, the results highlight that to create a detailed BC plan, different business divisions should integrate their tasks with the support of IT application teams (Tammineedi, 2010). This is needed to ensure the continuity and recovery of a company’s business operations to a predetermined acceptable level following a disruptive event (Sahebjamnia et al., 2015; Cerullo & Cerullo, 2004).

According to the findings, advanced IT governance practices receive the most weight, emphasising their importance in organisations. The Covid-19 pandemic has posed new challenges for many organisations. The current results show that changing an organisation’s needs complicates matters. As a result, using advanced technologies can help organisations stay competitive in this situation. The developed framework can help practitioners and managers overcome the challenges posed by the pandemic and remain competitive in the market during the difficult post-Covid-19 period (Pansare et al., 2022).

Also, based on the current study results, several managerial and practical implications are offered to companies’ board members, regulators, managers, and investors. Companies should have an effective and efficient IT governance structure and strategy. Members of the board, audit committees, other board committees, and external auditors should all be actively involved in IT governance and process. They should also establish an organisational IT governance structure and procedures that ensure explicit and strategic BC. IT governance should be at the top and a major focus of the board’s and audit committee’s agendas. Further, the effective participation of board and audit committee members could be secured by increasing board independence and expertise, which leads to effective monitoring and involvement of board members in IT governance. Board evaluation and training in IT governance issues are essential to avoid business disruption and achieve better BCM.

In times of emergency and economic crises, the behaviour of business organisations is critical. In crisis survival, firms’ resources, dynamic abilities, innovation, and practical strategies aid in combating the negative effects of the pandemic (Liu et al., 2021). Firms must be able to survive unprecedented threats, increase market exposure, and thrive on emerging opportunities in today’s volatile and fast-paced competitive business environment. Thus, IT plays a critical role in the success of modern organisations by influencing how they create and capture value (Mikalef et al., 2021). As a result, businesses have developed new strategies for surviving the Covid-19 pandemic. This is motivated by organisations striving for long-term viability through competitive activities (Liu et al., 2021).

Therefore, the Covid-19 economic crisis presented challenges and opportunities for marketing innovation and digitalisation to capitalise on business opportunities with competitive products to survive the crisis (Wang et al., 2020). Competitive firms enable business activities and provide opportunities to meet customer and business environment requirements that existed before the crisis but have increased during the Covid-19 era, such as additional services and digital solutions (Ilinova et al., 2021; Al-Hattami, 2021). As a result, the role of business firms’ innovativeness, resources at hand, business networks, and dynamic capabilities in producing the best products to compete in the business competition ultimately improves firm performance. These influential factors aid firms in surviving during emergencies and global crises (Liu et al., 2021).

Hence, effective IT governance would enable the implementation of decision-making structures and the efficient use of these resources to assist managers in achieving their strategic goals while minimising efforts and investments in IT (Frogeri et al., 2020). IT governance strengthens organizations’ resilience to potential economic and environmental shocks. Organisations, in particular, should improve their corporate governance in order to increase their resilience and survival in such a risky environment (Awwad & El Khoury, 2021). Business organisations hire high-tech employees to help with technological innovations that aid business success, organisational competitive advantage, and long-term survival. Consequently, this necessitates the implementation of new ideas generated by high-tech employees (Li et al., 2022).

### Limitations and directions for future research

Despite its numerous merits, this study contains several drawbacks. To begin, due to the length restriction of the questionnaire, the study was limited to a few corporate governance aspects. Thus, researchers are encouraged to investigate the other aspects of corporate governance that have not been considered here. Second, one major limitation this research encountered was collecting the data. Owing to the lockdown and Covid-19 restrictions, the study could not focus on particular sampling units. Third, this study is based on an emerging country, Jordan. Future research may investigate the same issues based on a comparison between some countries. Another limitation of this study is that it was conducted during Covid-19. A possible suggestion for future studies is to compare the findings with the post-Covid-19 situation. Finally, the current study is limited to a general sample drawn from different sectors. Future studies could compare several samples from different sectors.

## Data Availability

The data is available on request.
